# Supplementary material for: Metagenomic Insights Reveal the Microbial Diversity and Associated Algal-Polysaccharide-Degrading Enzymes on the Surface of Red Algae among Remote Regions
Source: Int J Mol Sci. 2023 Jul 3;24(13):11019. doi: 10.3390/ijms241311019 (PMC10342065; doi:10.3390/ijms241311019)
Supplement: Supplementary file 1 [file ijms-24-11019-s001.zip › ijms-2437135-supplementary.pdf]

Supplementary Material

Supplementary Tables

Table. S1 Data preprocessing statistics table

Sample ID	Insert size (bp)	Raw data	Clean data	Clean Q20	Clean Q30	Clean GC (%)	Effective (%)
NJDZ01	350	6,300.09	6,273.77	97.06	91.75	42.45	99.582
NJDZ02	350	6,159.60	6,135.73	97.19	91.92	42.30	99.612
NJDZ03	350	6,345.68	6,309.27	96.03	90.31	44.75	99.426
YNDZ01	350	12,079.83	12,023.87	97.58	93.41	42.08	99.537
YNDZ02	350	12,975.04	12,903.89	97.41	93.02	52.22	99.452
YNDZ03	350	12,258.91	12,165.99	97.03	92.56	43.43	99.242
WHDZ01	350	8,632.31	8,620.90	97.83	93.52	48.48	99.868
WHDZ02	350	8,508.37	8,470.83	98.05	94.31	55.78	99.559
WHDZ03	350	8,351.63	8,341.43	97.30	92.44	47.12	99.878

Table. S2 Assembly results of scaftigs statistics

Sample ID	Total len.(bp)	Num.	Average len.(bp)	N50 Len.(bp)	N90 Len.(bp)	Max len.(bp)
NJDZ01	290,645,811	261,344	1,112.12	1,174	568	67,860
NJDZ02	296,307,488	196,027	1,511.56	2,121	625	85,152
NJDZ03	400,006,748	237,800	1,682.11	2,421	708	60,595
YNDZ01	5,815,047	8,722	666.71	618	515	30,251
YNDZ02	692,737	1,098	630.91	596	512	2,077
YNDZ03	119,012,136	149,150	797.94	738	529	26,910
WHDZ01	230,893,252	205,409	1,124.07	1,190	573	514,363
WHDZ02	450,958,246	406,972	1,108.08	1,199	569	94,853
WHDZ03	360,929,708	362,829	994.77	1,021	559	64,501

Supplementary Figures

Supplementary Figure 1. The DNA quality control gel

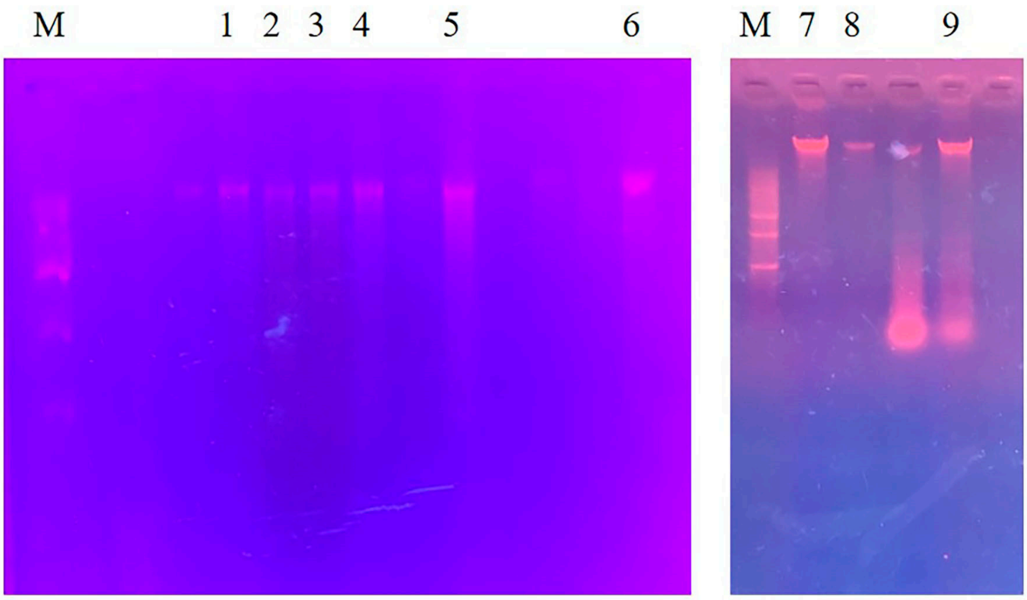

Note: M: DNA maker: 15000 bp, 10000 bp, 7500 bp, 5000 bp, 2500 bp, 1000 bp, 250 bp; 1: genomic DNA of *Palmaria decipiens*; 2: genomic DNA of *Curdiea racovitzae*; 3: genomic DNA of *Iridaea cordata*; 4: genomic DNA of *Laurencia japonica*; 5: genomic DNA of *Amphiroa foliacea*; 6: genomic DNA of *Gracilaria tenuistipitata; 7*: genomic DNA of *Grateloupia filicina*; 8: genomic DNA of *Chondrus ocellatus*; 9: genomic DNA of *Hyalosiphonia caespitosa*

Supplementary Figure 2. Annotation of EggNOG functional taxa

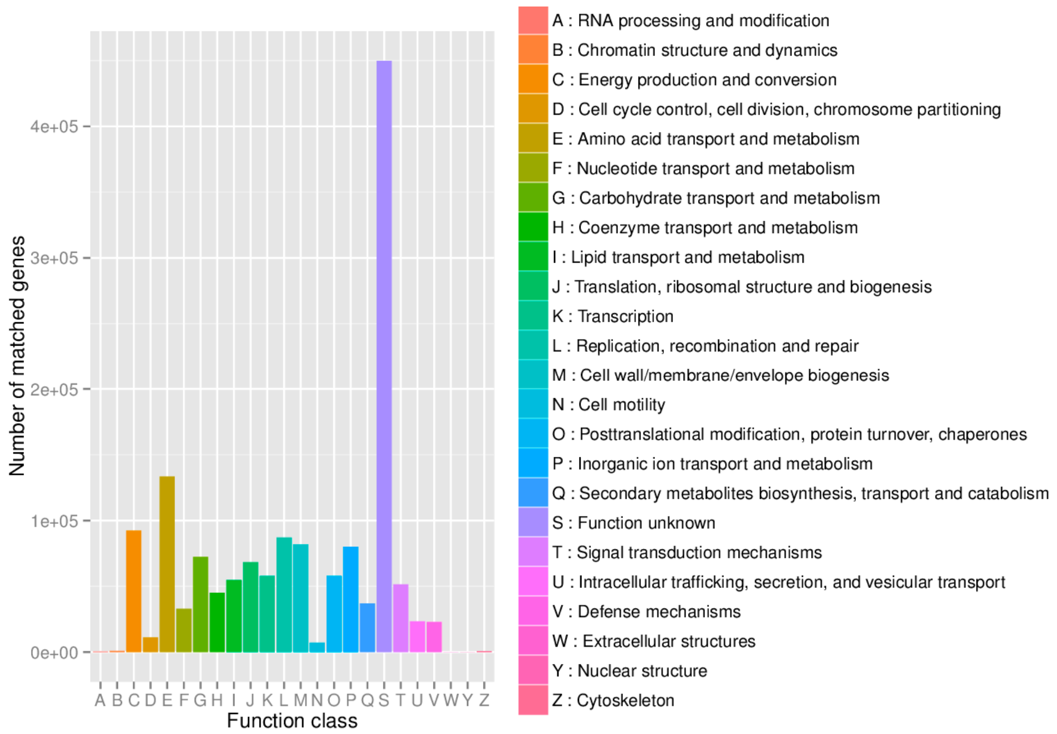

Fig. S2

Supplementary Figure 3. Abundance of different antibiotic resistance ontology in each sample

a: the relative abundance of all genes of ARO in each sample was amplified 10^6 times by the original relative abundance data; b: top20 relative abundance of AROs among all AROs.

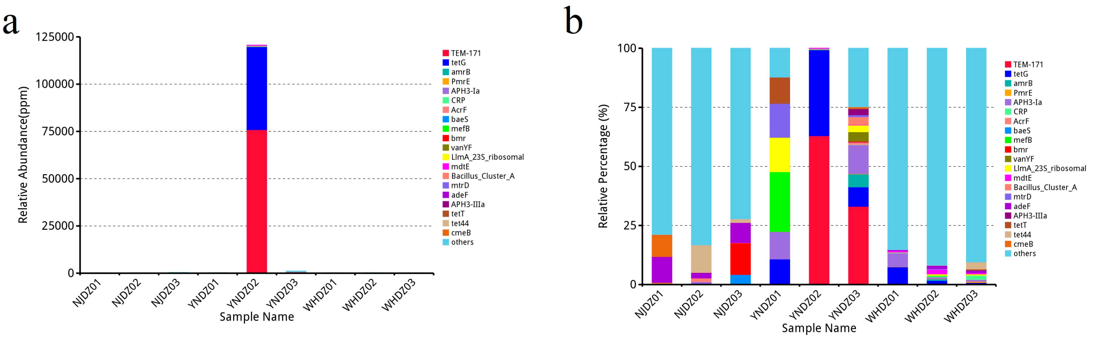

Fig. S3
